# Mortality rate in patients in a long-term psychiatric care facility in Johannesburg

**DOI:** 10.4102/sajpsychiatry.v30i0.2329

**Published:** 2024-11-27

**Authors:** Mokgokong Mathekga, Nokhutula Mdaka, Mvuyiso Talatala

**Affiliations:** 1 Department of Psychiatry, Faculty of Health Sciences, University of the Witwatersrand Johannesburg, South Africa; 2 Department of Psychiatry, Faculty of Health Sciences, Chris Hani Baragwanath Academic Hospital, University of the Witwatersrand, Johannesburg, South Africa

**Keywords:** long-term care, severe mental illness, mortality, schizophrenia, standardised mortality ratio

## Abstract

**Background:**

Limited research exists on mortality rates and contributing factors among individuals with severe mental illness (SMI) in long-term psychiatric care, especially in low- and middle-income countries (LMICs).

**Aim:**

To analyse mortality rates and associated factors at Solomon Stix Morewa Memorial Hospital (SSMMH), a long-term psychiatric care facility in Johannesburg, South Africa.

**Setting:**

SSMMH, a private facility in Selby Park, Johannesburg, has been contracted by the Gauteng Department of Health since March 2017 to provide inpatient care for SMI patients.

**Methods:**

A retrospective analysis was conducted on records of 406 SMI patients admitted to SSMMH from March 2017 to February 2022. Mortality rates were calculated, and associated factors were analysed using descriptive statistics and logistic regression.

**Results:**

Of the 406 participants, 34 (8%) died over five years, yielding a standardised mortality ratio (SMR) of 1.28 (95% CI: 0.85–1.71). Mortality was highest in the 60–69 years age group (SMR 2.7), with most deaths occurring in 2020–2021, likely due to COVID-19. Cardiovascular conditions were the leading cause of death (53%). Age at admission (OR: 2.35, 95% CI: 1.55–3.58) and transfer site (OR: 0.58, 95% CI: 0.42–0.81) were significant predictors of mortality.

**Conclusion:**

Individuals with SMI face elevated mortality rates, with age, diagnosis, and comorbidities as key factors.

**Contribution:**

This study provides insights into the comprehensive management of people with SMI to reduce mortality. Further research is needed to guide psychosocial and palliative care approaches.

## Introduction

High mortality in people with severe mental illness (SMI) such as schizophrenia has been clearly shown in many studies.^1,2^ Patients with SMI have a life expectancy that is 15–20 years shorter than that of the general population.^3^ This reduced life expectancy in people with SMI contrasts with the recent trend of increased longevity in the general population. As a result, there is a substantial mortality gap between people with mental illness and the general population.^4^

A recent systematic review and meta-analysis further confirmed that patients with schizophrenia have much higher mortality than the general population. In this study, patients with schizophrenia had a mortality risk ratio of 2.94 when compared to the general population.^2^ Additionally, the mortality risk of patients with schizophrenia was still significant when compared with controls who had other medical conditions. Most studies in this systematic review and meta-analysis were mainly from high-income countries. However, similar findings have been reported in low- and middle-income countries (LMICs). In a 5-year outpatient follow-up study of a cohort of patients with schizophrenia in a rural setting in Ethiopia by Teferra et al., the overall standard mortality ratio (SMR) was 5.98.^5^ Contrary to this finding, Khamker et al. did not find a significant SMR in patients who were hospitalised in a psychiatric hospital in South Africa from 2001 to 2005.^6^ Khamker et al. hypothesised that this unusual finding – mortality in people with SMI not being significantly higher than the general population – was because of the acquired immunodeficiency syndrome (AIDS) pandemic that caused excess mortality in the general population of South Africa.

In South Africa’s Gauteng province, palliative psychiatric care is provided in hospital settings known as long-term care facilities (LTCFs). This care is in addition to community-based mental health services that include community residential care provided by non-governmental organisations (NGOs). Robertson and Makgoba conducted a mortality analysis on a group of patients in Johannesburg who were rapidly discharged from LTCFs to community-based NGOs that were ill-equipped to care for patients with SMI.^7^ In their analysis, they found that 9% of patients had died between March 2016 and April 2017, with an overall age-adjusted death rate of 63/1000 population. The overall SMR in this study population was 4.9.

The SMR is an important epidemiological metric used to assess the relative increase or decrease in mortality within a study cohort compared to the general population. It is typically represented as a ratio or percentage, where an SMR of 1.0 indicates equality between observed and expected deaths. Standard mortality ratios exceeding 1.0 indicate a higher mortality rate than expected in the study group, while values below 1.0 suggest fewer deaths than anticipated.^8,9^ The SMR is a useful measure of mortality because it considers how demographic factors, environmental factors, socioeconomic status, healthcare access and specific diseases can impact mortality within a population.^10^ Standard mortality ratios in people with SMI have increased significantly during the 1970s, 1980s and 1990s, from 1.84 to 2.98 to 3.20, respectively, compared to the general population and further confirms the increased mortality in people with SMI.^11,12^

The causes of mortality in SMI include non-natural causes such as suicide and injuries as well as natural causes such as cardiovascular and related causes, diabetes mellitus, infectious diseases and lifestyle-related causes. In a 12-year cross-sectional study conducted in Ontario, Canada, cardiovascular-related diseases (CVDs) were the main cause of death in patients with psychotic disorders followed by neoplastic, metabolic and respiratory causes.^13^ A 24-year retrospective registry study conducted in Sweden revealed that individuals with schizophrenia had a threefold higher mortality rate from CVDs compared to the general population.^14^

Lifestyle factors are directly linked to cardiovascular complications that are found in patients with SMI. People with schizophrenia have reduced physical activity, increased smoking and make poor dietary choices which all increase the risk of cardiovascular diseases as well as the risk of neoplasms.^11,15,16^

While there is rich literature on mortality in people with SMI from high income countries, there is a notable paucity in research with regards to the mortality rate in patients with SMI in LMICs such as South Africa. The existing South African literature has focussed on mortality among people living with SMI in hospital setting as well as the findings by Robertson and Makgoba in NGOs.^7^ To our knowledge, there has not been a mortality analysis in patients with SMI admitted in a LTCF. The objective of this study was to determine the annual mortality rate and overall mortality rate in psychiatric patients admitted at Solomon Stix Morewa Memorial Hospital (SSMMH), a LTCF in Johannesburg, South Africa, during the period from 01 March 2017 to 28 February 2022. Additionally, the study described the socio-demographic profile, the psychiatric data and other medical data of all the patients who were admitted at SSMMH during this study period.

## Research methods and design

### Study design and site

This was a quantitative retrospective cross-sectional study which was conducted at SSMMH. The SSMMH is a private facility in Selby Park, Johannesburg, that has been contracted by the Gauteng Provincial Department of Health since March 2017 to provide inpatient chronic psychiatric care for patients with SMI.

The overall and annual mortality rate at SSMMH was calculated and compared with the mortality rate of the general population. The mortality rate of the general population of South Africa was obtained from Statistics South Africa.^17^ The study also analysed how different demographic factors and medical and psychiatric factors are associated with mortality rates.

### Study population and sampling

The study included all psychiatric patients admitted at SSMMH for LTCF services from 01 March 2017 to 28 February 2022. The study participants, totalling 406 individuals, were transferred to NGOs and public hospitals from the southern and western regions of Gauteng. The patients that were from NGOs are the patients who were rapidly discharged from a LTCF, Life Esidimeni (LE), to the ill-equipped NGOs.^18^ Participants who were admitted outside the specified study period were excluded.

### Data collection

Data were collected using the patients’ files and a structured data collection sheet. The collected data were then transferred to a Microsoft Excel™ spreadsheet for further analysis. To ensure confidentiality, a unique code was assigned to each file. The data collected from the files comprised of demographic details including age, gender and education level, along with medical information such as diagnoses of medical conditions, coronavirus disease 2019 (COVID-19) test results and psychiatric data including mental illness diagnoses, treatment regimens, and causes of death.

### Statistical analysis

A descriptive analysis was conducted on socio-demographic profile, psychiatric and medical information, and causes of death. Categorical data such as causes of death and gender were summarised using frequencies and percentages, *n* (%). Numeric variables, such as patients’ average time to death, were summarised using central tendency measures, such as the mean and standard deviation (s.d.). Pearson Chi-square test was conducted to determine the association between demographic, physical and medical factors among patients who died during the review period. A logistic regression analysis was used to determine the effects of dependent (causes of death) and independent variables (demographic and medical information). Odds ratios (OR) and 95% confidence interval (CI) were reported, and the Alpha value < 0.05 was considered statistically significant for unadjusted comparisons. The analysis was conducted using the statistical package SAS, version 9.4 (SAS Institute, Cary, North Carolina, United States).

### Ethical considerations

Ethics approval was granted by the University of Witwatersrand Human Resources Ethics Committee (clearance certificate M221011). Permission to conduct the study was obtained from the Clinix Health Group and the hospital manager at SSMMH. To maintain confidentiality, patient identifying data were excluded by using a unique identifying code in the data set.

## Results

### Demographic characteristics

During the study period (01 March 2017 to 28 February 2022), 406 participants were admitted to SSMMH. Most participants were former LE patients who were transferred to NGOs and later admitted at SSMMH (*n* = 318, 78%). The rest were transferred from hospitals in Gauteng (*n* = 88, 22%) (Table 1).

**TABLE 1:** Summary statistics for mortality by transfer facility.

Transfer Facility	Mortality No	Yes	Total	% transferred	Mortality relative to population transferred (%)
CHBAH	23	1	24	6	4
CMJAH	5	1	6	1	20
HJH	7	0	7	2	0
NGOs (Former LE patients)	286	32	318	78	46
SFH	36	0	36	9	0
South rand hospital	6	0	6	1	0
Tara h moross hospital	9	0	9	2	0

**Total**	**372**	**34**	**406**	**-**	**-**

CHBAH, Chris Hani Baragwanath Hospital; CMJAH, Charlotte Maxeke Academic Hospital; HJH, Helen Joseph Hospital; SFH, Sterkfontein Hospital.

The demographic profile of the participants was as follows: majority were males (*n* = 327, 81%), most were single (*n* = 397, 98%) and in the 50–59 years age group (*n* = 124, 33%). All the participants were unemployed, and half of the participants had a high school education (Table 2).

**TABLE 2:** Demographic characteristics of study population.

Demographic characteristic	Total number: 406	%	Death Total number: 34		*P*
			*n*	%	
**Gender**					
Female	79	19	13	38	0.004
Male	327	81	21	62	-
**Age group (years)**					
18–29	16	4	-	-	*P* < 0
30–39	75	18	1	3	-
40–49	110	27	6	18	-
50–59	134	33	10	29	-
60–69	54	13	12	35	-
70–79	12	3	3	9	-
80 and above	5	1	2	6	-
**Marital status**					
Married	2	0	-	-	0.957
Single	398	98	34	100	-
Widow/Widower	3	1	-	-	-
Unknown	2	0	-	-	-
**Level of education**					
Higher education	17	4	-	-	0.318
High school	203	50	21	62	-
Primary school	97	24	7	21	-
Special school	19	5	3	9	-
None	13	3	1	3	-
Unknown	57	14	2	6	-

### Psychiatric disorders and other medical comorbidities

In terms of the psychiatric diagnosis, majority of the participants had a diagnosis of schizophrenia (*n* = 336, 86%); this was followed by the diagnosis of bipolar and related disorders (*n* = 31, 8%) (Table 3). The prevalence of neurocognitive disorders and major depressive disorder was low.

**TABLE 3:** Summary statistics: Psychiatric disorders.

Psychiatric disorders	Study population (*N* = 406)		Death (Yes)	
	*n*	%	*n*	%
Schizophrenia	336	83	33	97
Bipolar and related disorders	32	8	1	3
Mental illness secondary to another medical condition	17	4	-	0
Neurodevelopmental disorders	13	3	-	0
Neurocognitive disorder	7	2	-	0
Depressive disorders	1	0	-	0

**Grand total**	**406**	**100**	**34**	**100**

Note: Count and percentage data are shown.

With regards to other medical comorbidities, 66% (*n* = 266) of the participants had a comorbid medical condition, with hypertension (*n* = 154, 38%) being the most common medical condition followed by type 2 diabetes mellitus (*n* = 73, 18%).

### Mortality rate

**FIGURE 1:**
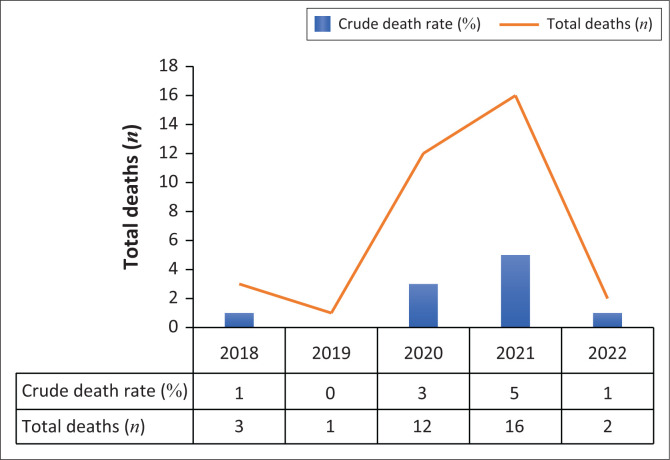
Crude annual mortality rates and number of deaths by year at Solomon Stix Morewa Memorial Hospital.

Of the 406 participants, 34 (8%) died during the 5-year study period (Table 2). The SMR for the 5-year study period was 1.28 (95% CI = 0.85–1.71) (Table 4). The annual SMR was not calculated as there were very few deaths recorded at SSMMH in 2018 (*n* = 3), 2019 (*n* = 1) and 2022 (*n* = 2) (Figure 1).

**TABLE 4:** Standard mortality ratio according to age group.

Age groups	Death rate per 1000 in Population	SSMMH Population	Expected deaths at SSMMH	Observed deaths at SSMMH	SMR in different age groups	95% confidence interval
18–29	30	16	0.48	0	0.00	(0.0 – 0.0032)
30–39	60	75	4.50	1	0.22	(0.0 – 0.65)
40–49	60	110	6.60	6	0.90	(0.18 – 1.64)
50–59	70	134	9.40	10	1.06	(0.39 – 1.73)
60–69	84	54	4.50	12	2.67	(1.16 – 4.18)
70–79	72	12	0.86	3	3.49	(0 – 7.43)

**Total**	**-**	**406**	**26.58**	**34**	**-**	**-**

Note: Standard population rate taken from Stats SA.^19^

SMR = number of observed deaths in study population/numbers of expected deaths if mortality rate was the same as general population; SMR = 34/26.58 =1.28.

It was established that nearly twice (*n* = 21, 62%) as many males died compared to females (*n* = 13, 38%). While the total number of males who died is higher than females, it must be noted that our study population had a higher proportion of males (81%) than females (19%). As a result, the percentage of deaths was higher among females, with 16% (13 of 79) of female participants in the study population having died, compared to 6% (21 of 327) of male participants during the study period.

The highest number of deaths were in the 60–69 years age group (*n* = 12, 35%) followed by the 40–49 years and 50–59 years age groups, accounting for 18% (*n* = 6) and 29% (*n* = 10) of the deaths, respectively (Table 2). From the 34 recorded deaths, the period between 2020 and 2021 accounted for 82% of the total deaths (Figure 1). More specifically, nearly half of these deaths occurred in 2021, representing 47% of the total deaths, while just over a third occurred in 2020, accounting for 35% of the total deaths.

**FIGURE 2:**
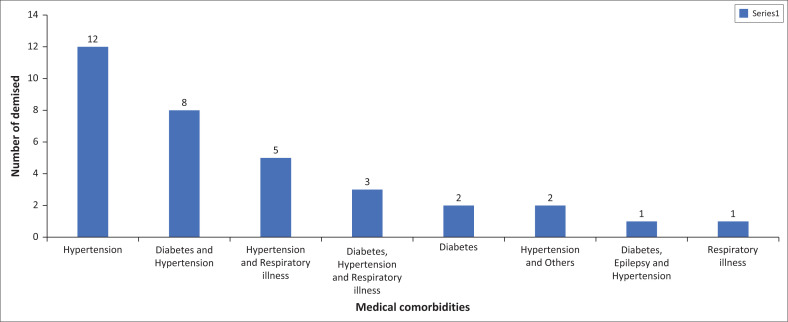
Medical comorbidities in participants who died.

All 34 participants who died had a medical comorbidity with hypertension being the most prevalent medical comorbidity (*n* = 12, 35%), and 24% (*n* = 8) participants had a combination of hypertension and diabetes (Figure 2).

### Interpretation of logistics regression results

The findings revealed that age at admission, relationship status and transfer site were significant predictors of mortality.

The OR for age at admission was 2.35 95% CI (1.55 – 3.58) and for every 1-year increase in age, the odds are 2.35 higher, signifying that individuals of advanced age were more prone to mortality compared to younger patients. Furthermore, mental health illness, although marginally significant (*p* = 0.06), played a noteworthy role in predicting mortality.

## Discussion

The overall mortality rate in this study was 8%, with a SMR of 1.28 (95% CI = 0.85–1.71). This indicates significantly high mortality among individuals with SMI at SSMMH compared to the general population, consistent with the broader literature.^7,12,20^ Of significance is that this SMR was lower than that found by Robertson and Makgoba in a similar cohort of patients but in a different setting (community-based NGOs vs. LTCF).^7^ The mortality analysis by Robertson and Makgoba was done in a setting that was characterised by poor quality of care.^21^ It can therefore be inferred that patients with SMI need to be cared for in an appropriate setting as they present with complicated psychiatric disorders and medical comorbidities. Interestingly, the SMR of 1.28 observed in this study was also lower than those reported in other local and global studies, which have ranged from 2.58 to 5.98.^6,22^ Possible explanations for the relatively lower SMR include the comprehensive care model at SSMMH, with full-time physicians collaborating closely with psychiatrists and dedicated nursing staff to ensure prompt medical treatment and medication adherence. Additionally, the high utilisation of anti-psychotic medications, including clozapine, which has been associated with a 30% – 50% reduced mortality risk compared to non-use, may have contributed to the lower mortality observed in this cohort.^23^

High mortality rates were seen in the older age group of 60–69 years (SMR 2.7) and among the female participants. Other studies have reported similar findings in terms of the SMR in this age group.^2,24,25,26^ While mortality is expected to increase with ageing, it is noteworthy that the mortality in older people with SMI is higher than in the general population. Severe mental illness is associated with accelerated ageing because of multiple factors such as inflammation, lifestyle factors, genetic factors and there is also evidence of accelerated brain ageing.^27^

The high mortality rate among females is in keeping with the findings from the study conducted by Robertson and Makgoba with a SMR of 3.9 for men and 6.3 for women. Contrary to this finding, other studies have however reported a higher mortality rate among males.^5,28^ In a study conducted by Nhiwatiwa et al. on the occurrence of hyponatraemia in patients with SMI at SSMMH, a similar cohort to the current study, it was found that 52.1% of the females had hyponatraemia at least once during the admission, while only 26.3% of the males had hyponatraemia at least once in their admission.^29^ It is likely that there are specific factors that increased vulnerability of females to mortality in this cohort of patients, and hyponatraemia could be one of those factors.

While annual SMR could not be calculated in this study, there was high crude mortality rate in the years 2020 (3%) and 2021 (5%) compared to the years 2018, 2019 and 2022, with 2019 having no recorded death at SSMMH. Most deaths occurred in the years 2020 and 2021 (82%) at SSMMH. This increased mortality in these 2 years is likely because of COVID-19 pandemic which had its highest impact in terms of mortality in the years 2020 to 2021 in South Africa.^30,31^ It worth noting that this increase in mortality at SSMMH in 2020 and 2021 did bring SMR at SSMMH to the high levels that have been reported in literature which is further evidence that the increased mortality at SSMMH in these years was accompanied by excess deaths in the general population.

Similar to other studies, key contributors to mortality in this cohort included advanced age, a diagnosis of schizophrenia and the presence of medical comorbidities such as hypertension or type 2 diabetes.^2,3,14,32^ People with schizophrenia have increased mortality and have a life expectancy 15–20 years shorter than the general population.^12,33,34,35^ Proposed factors that are associated with the increased mortality in schizophrenia include the high rates of suicide, homicide and other unnatural causes, as well as the impact of cognitive decline and substance use on vulnerability and healthcare access.^33,36^ There is an approximately 10% lifetime risk of suicide in people with schizophrenia.^37^

However, suicide as a cause of death was not reported in this study.

The study findings revealed a strong association between medical comorbidities and mortality rates. Approximately 66% of participants presented with comorbid medical conditions, predominantly hypertension (38%) and type 2 diabetes (18%). Notably, every deceased participant had at least one medical comorbidity, most commonly hypertension (35%) and diabetes (18%). This pattern of increased prevalence of cardiometabolic conditions among individuals with mental illness has been consistently observed across various studies.^34^ The elevated rates of hypertension and diabetes in schizophrenia can be attributed to a multitude of factors, including sedentary lifestyles, poor dietary habits, substance use and the metabolic side effects of anti-psychotic medications.^34^ A local South African study conducted in KwaZulu-Natal further corroborated these findings, showing that one-fifth of patients with SMI on anti-psychotics met the criteria for metabolic syndrome, with a higher prevalence among women and the elderly.^38^

Compounding this burden, individuals with mental illness often receive sub-optimal medical care because of stigma, a lack of healthcare education and limited access to services, leading to inadequate management of cardiometabolic conditions and associated complications.^39,40,41^ These complex factors contribute to the high prevalence of medical comorbidities and their adverse impact on mortality in this vulnerable population.

Cardiovascular-related conditions were the leading cause of death, accounting for over half (53%) of the mortalities (see Table 5).This finding is in keeping with the existing literature, which identifies cardiovascular diseases as the primary driver of mortality in individuals with schizophrenia.^42^ Other studies have reported that among individuals with SMI, preventable physical illnesses, particularly cardiovascular diseases, respiratory conditions and infections, account for most fatalities.^43,44^ In a study by de Barros et al., respiratory-related conditions were the second leading cause of death, responsible for 35% of the mortalities, potentially exacerbated by factors such as overcrowded psychiatric wards, poor hygiene and high smoking rates among this population.^45^

**TABLE 5:** Causes of mortality.

Cause of death	Death (yes)	Total (%)
Cardiovascular related	18	53
Respiratory related	12	35
GIT related	3	9
Respiratory related, neoplastic related	1	3
Other	-	-

**Total**	**34**	**100**

Note: Count and percentage data are shown.

GIT, gastrointestinal tract.

### Limitations of the study

This was a retrospective study that relied on the quality of record keeping by the clinical staff at SSMMH and the hospital record keeping. The attribution of COVID-19 as a causative factor for mortality in the years 2020 and 2021 is inferred as the records did not accurately label the deaths in these years as because of COVID-19 infection. Lastly, the inherent difficulty in controlling for all potential confounding variables presents a challenge in establishing a precise relationship between the exposure and outcome.

### Implications and future directions

In summary, this study underscores the critical need to recognise and address factors contributing to the high mortality rate in individuals with SMI, particularly those diagnosed with schizophrenia. The findings emphasise the importance of targeted interventions, improved healthcare access and comprehensive care strategies to mitigate the impact of both mental and physical health challenges in this vulnerable population.

This study emphasises the ongoing necessity for further research focussing on the mortality rate of people with SMI. Well-designed prospective studies are necessary to gain a deeper understanding of the complex interplay of factors influencing mortality in this population. Additionally, there is a need for more studies to explore mortality in mental illness within the South African context. A follow-up study on the mortality rate in the post-COVID-19 era should be conducted to assess any changes or impacts. Furthermore, allocating more resources to research and introducing palliative psychiatric care for individuals who require LTCFs but do not have remitting psychiatric symptoms are essential.

## Conclusion and recommendations

This study confirms that people with SMI have increased mortality rates. Various factors, such as older age, female gender, specific psychiatric diagnoses and the existence of medical comorbidities, exhibit positive correlations with an elevated risk of mortality within this population. From this study, it can be hypothesised that placement in a LTCF offers some protective factors than being in a poorly resourced NGO.

### Recommendations

- More studies to explore mortality in mental illness in the South African context. A follow-up study of the mortality rate in ‘the post-COVID-19 era’ should be conducted.
- More resources in research and introduction of palliative psychiatric care for individuals who need LTCF but without remitting psychiatric symptoms.
- Research in palliative psychiatry programmes that can be implemented in LTCFs, NGOs and in other mental health care facilities in the community.
